# Neuroimaging and Clinical Findings in Healthy Middle-Aged Adults With Mild Traumatic Brain Injury in the PREVENT Dementia Study

**DOI:** 10.1001/jamanetworkopen.2024.26774

**Published:** 2024-08-15

**Authors:** Audrey Low, Elizabeth McKiernan, Maria A. Prats-Sedano, Stephen F. Carter, James D. Stefaniak, Li Su, Maria-Eleni Dounavi, Graciela Muniz-Terrera, Natalie Jenkins, Katie Bridgeman, Karen Ritchie, Brian Lawlor, Lorina Naci, Paresh Malhotra, Clare Mackay, Ivan Koychev, Tony Thayanandan, Vanessa Raymont, Craig W. Ritchie, William Stewart, John T. O’Brien

**Affiliations:** 1 Department of Psychiatry, School of Clinical Medicine, University of Cambridge, Cambridge, United Kingdom; 2 Department of Clinical Neurosciences, University of Cambridge, Cambridge, United Kingdom; 3 Department of Neuroscience, University of Sheffield, Sheffield, United Kingdom; 4 Edinburgh Dementia Prevention, University of Edinburgh, Edinburgh, United Kingdom; 5 School of Psychology and Neuroscience, University of Glasgow, Glasgow, United Kingdom; 6 Department of Neuropathology, Queen Elizabeth University Hospital, Glasgow, United Kingdom; 7 INSERM, Montpellier, France; 8 Institute of Neuroscience, Trinity College Dublin, University of Dublin, Dublin, Ireland; 9 Division of Brain Science, Imperial College Healthcare NHS Trust, London, United Kingdom; 10 Department of Psychiatry, Oxford University, Oxford, United Kingdom; 11 Scottish Brain Sciences, Edinburgh, United Kingdom; 12 Cambridgeshire and Peterborough NHS Foundation Trust, Cambridge, United Kingdom

## Abstract

**Importance:**

Traumatic brain injuries (TBI) represent an important, potentially modifiable risk factor for dementia. Despite frequently observed vascular imaging changes in individuals with TBI, the relationships between TBI-associated changes in brain imaging and clinical outcomes have largely been overlooked in community cases of TBI.

**Objective:**

To assess whether TBI are associated with and interact with midlife changes in neuroimaging and clinical features in otherwise healthy individuals.

**Design, Setting, and Participants:**

This cross-sectional analysis used baseline data from the PREVENT Dementia program collected across 5 sites in the UK and Ireland between 2014 and 2020. Eligible participants were cognitively healthy midlife adults aged between 40 and 59 years. Data were analyzed between January 2023 and April 2024.

**Exposure:**

Lifetime TBI history was assessed using the Brain Injury Screening Questionnaire.

**Main Outcomes and Measures:**

Cerebral microbleeds and other markers of cerebral small vessel disease (white matter hyperintensities [WMH], lacunes, perivascular spaces) were assessed on 3T magnetic resonance imaging. Clinical measures were cognition, sleep, depression, gait, and cardiovascular disease (CVD) risk, assessed using Computerized Assessment of Information Processing (COGNITO), Pittsburgh Sleep Quality Index, Center for Epidemiologic Studies Depression Scale, clinical interviews, and the Framingham Risk Score, respectively.

**Results:**

Of 617 participants (median [IQR] age, 52 [47-56] years; 380 female [61.6%]), 223 (36.1%) had a history of TBI. TBI was associated with higher microbleed count (β = 0.10; 95% CI, 0.01-0.18; *P* = .03), with a dose-response association observed with increasing number of TBI events (β = 0.05; 95% CI, 0.01-0.09; *P* = .03). Conversely, TBI was not associated with other measures of small vessel disease, including WMH. Furthermore, TBI moderated microbleed associations with vascular risk factors and clinical outcomes, such that associations were present only in the absence of TBI. Importantly, observations held when analyses were restricted to individuals reporting only mild TBI.

**Conclusions and Relevance:**

In this cross-sectional study of healthy middle-aged adults, detectable changes in brain imaging and clinical features were associated with remote, even mild, TBI in the general population. The potential contribution of vascular injury to TBI-related neurodegeneration presents promising avenues to identify potential targets, with findings highlighting the need to reduce TBI through early intervention and prevention in both clinical care and policymaking.

## Key Points

**Question:**

Does remote traumatic brain injury (TBI) history relate to observable changes in neuroimaging and clinical features in a general population of middle-aged adults?

**Findings:**

In this cross-sectional investigation of 617 healthy middle-aged adults, history of even mild TBI was associated with detectable changes in neuroimaging and clinical outcomes. Participants with prior TBI experienced poorer sleep and depressive symptoms, but with no significant change in cognition.

**Meaning:**

These findings underscore the potential long-lasting brain health consequences of even mild TBI, with the prospect that these might be detectable long before expected clinical manifestations of related neurodegenerative disease, presenting a target for risk stratification and intervention.

## Teaser

This cross-sectional study of middle-aged US adults examines the association of traumatic brain injury (TBI) with neuroimaging and clinical outcomes.

## Introduction

Traumatic brain injury (TBI) is increasingly recognized as an important and modifiable risk factor for dementia.^1^ However, the biological processes driving an acute biomechanical injury to a chronic, lifelong pathology associated with late neurodegenerative disease remain uncertain.^2,3^ Among TBI-related pathologies, vascular injury is recognized as a common pathology, with outcomes ranging from macrovascular or microvascular hemorrhage to blood-brain barrier disruption.^4,5,6^ Studies on TBI-related neuroimaging changes have largely focused on contact sports athletes (eg, American football players, professional fighters) or inpatient trauma units,^7,8,9^ which tend to be limited by smaller sample sizes and have higher exposure or severity of TBI. Conversely, TBI-related changes in brain imaging and clinical outcomes have largely been overlooked in the everyday context of healthy midlife adults, especially in relation to mild TBI.

Cerebral microbleeds (CMB) reflect hemosiderin deposition resulting from microvascular injury^10,11,12,13^ and are considered key markers of cerebral small vessel disease (SVD).^14,15^ However, CMB can also arise as a consequence of TBI, which is often overlooked in research exploring their clinical and prognostic utility.^5^ Specifically, TBI as a contributor to CMB is rarely considered in the study of SVD, which could potentially explain why the clinical correlates of SVD are not consistently demonstrated in CMB research.^10,16,17,18^As a key marker of SVD, one may reasonably expect CMB to relate to well-established correlates of SVD itself, such as vascular risk factors (eg, hypertension) and clinical manifestations of SVD like cognitive impairment, gait disturbances, depression, and disturbed sleep.^19,20^ Therefore, the inconsistent and often nonexistent link between CMB and these established variables presents an apparent contradiction that warrants deeper investigation.^5^

To address these uncertainties, we leverage comprehensive imaging, clinical, and sociodemographic data of otherwise healthy individuals to investigate the associations of lifetime TBI history with early (midlife) SVD and clinical features (aim 1), and the possible influence of TBI history on the associations between CMB and measures of vascular risk factors and clinical deficits (aim 2). We hypothesized that these associations would be weaker in individuals with TBI, given that TBI-induced CMB may result as a consequence of head trauma rather than underlying microvascular disease.^21,22,23,24^

## Methods

### Participants

Participants were recruited as part of the PREVENT Dementia program^25^ (study protocol published elsewhere).^26,27,28,29^ Participants had to be cognitively healthy (no dementia or other neurological conditions) middle-aged adults (aged 40 to 59 years). Of the 700 recruited, 617 were included based on completeness of imaging and TBI data, and absence of incidental magnetic resonance imaging (MRI) findings (eFigure 1 in Supplement 1). Data were collected across 5 sites in the UK and Ireland between 2014 and 2020, and analyzed between January 2023 to April 2024.

The study was approved by the London-Camberwell St Giles National Health Service Ethics Committee, which operates according to the Helsinki Declaration of 1975.^30^ All participants gave written informed consent. This study follows the Strengthening the Reporting of Observational Studies in Epidemiology (STROBE) reporting guideline.

### Quantification of Cerebral Small Vessel Disease

Imaging markers of SVD were assessed on 3T MRI (Siemens; acquisition parameters in eAppendix of Supplement 1) according to the Standards for Reporting Vascular Changes on Neuroimaging (STRIVE) guidelines.^14^ CMB were assessed on 3T susceptibility-weighted imaging scans using the Microbleed Anatomical Rating Scale (MARS),^31^ and cross-validated on T1- and T2-weighted images. Lacunes were assessed on T1-weighted, T2-weighted, and FLAIR images.^14^ Lacunes and CMB were classified by location as deep or lobar. WMH volumes were extracted from lesion maps created on FLAIR MRI using an automated script on SPM12.^32^ All WMH maps were visually inspected and manually corrected for misclassifications, and WMH volumes were normalized by total intracranial volume. Perivascular spaces (PVS) were assessed separately in the basal ganglia (BG) and centrum semiovale (CSO) on T2-weighted scans using a validated rating scale.^33^ Raters were masked to all clinical information, including TBI history. Further details on SVD quantification and interrater reliability evaluation can be found in the eAppendix in Supplement 1 and previous publications.^28,34^

### Clinical and Neuropsychological Assessment

Cognitive performance was assessed using the Computerized Assessment of Information Processing (COGNITO) battery.^35^ Composite scores for each cognitive domain were computed by averaging the *z*-scores of relevant tasks in each domain as previously defined (eAppendix in Supplement 1).^35,36^ Depression was measured using the Center for Epidemiologic Studies Depression Scale (CES-D).^37^ Quality of sleep was measured with the Pittsburgh Sleep Quality Index (PSQI).^38^ For the CES-D and PSQI, higher scores indicate greater depressive symptoms and sleep disturbances, respectively (eAppendix in Supplement 1). Gait disturbances were assessed as part of neurological examinations by a qualified clinician and assigned binary classifications (normal, abnormal). Alcohol intake was dichotomized using a cut-off of more than 21 units per week.

### Assessment of Traumatic Brain Injury

History of TBI was assessed using the Brain Injury Screening Questionnaire (BISQ).^39^ The BISQ includes structured recall cues to aid individuals’ recall of injuries, using 20 scenarios where head injuries may have occurred with a further option to record injuries not otherwise listed. For each item endorsed, participants are asked whether this resulted in a loss of consciousness (LOC) or being dazed and confused, and for how long. Participants with TBI were defined as those experiencing at least 1 blow to the head resulting in LOC, with mild TBI (mTBI) defined where LOC was less than 30 minutes (eAppendix in Supplement 1).^40^

### Assessment of Cardiovascular Risk

Risk of cardiovascular disease (CVD) was assessed using the Framingham Risk Score,^41^ an established tool for estimating 10-year cardiovascular risk using a sex-specific multivariable algorithm. The Framingham Risk Score is based on age, systolic blood pressure (BP), use of antihypertensive medication, current smoking, diabetes, total cholesterol, and high-density lipoprotein cholesterol (HDL). Computation and cut-offs used to derive the Framingham Risk Score are adapted from Wilson and colleagues.^41^

### Statistical Analysis

Standard statistical techniques were used for descriptive analyses (Table 1; eAppendix in Supplement 1). Unless otherwise stated, all regression models adjusted for sex, age, education, and study site. Models were constructed to assess (1) TBI and other risk factors associated with SVD, (2) the association of TBI with clinical and imaging measures, (3) if TBI may moderate the association between CMB and expected clinical correlates, and (4) the relative contribution of TBI and vascular risk factors to clinical deficits in the subset of participants with TBI. For how TBI and other risk factors (age, CVD risk, hypertension, apolipoprotein E ε4 [APOE4]) are associated with SVD, adjusted regression models were independently fitted in separate models with risk factors as exposure variables and SVD markers as outcome variables (included in aim 1). To examine how TBI related to clinical and imaging measures, adjusted regression models were fitted to markers of SVD (WMH volume, PVS, CMB, lacunes) and clinical measures (PSQI, gait disturbances, CES-D score of depression, cognition) with TBI as the exposure variable (aim 1). To investigate whether TBI moderated associations between CMB and expected clinical correlates, interaction analysis was conducted with *CMB* × *TBI* interaction terms fitted to outcome variables (eg, cognition, CVD risk) (aim 2). To compare the relative contribution of TBI and vascular risk factors to clinical deficits in the subset of participants with prior TBI, dominance analysis and relative weights analysis were conducted (eAppendix in Supplement 1).^42,43^ Outcome variables were clinical deficits (poor sleep, gait disturbances, depressive symptoms, cognition) while exposure variables were TBI (number of TBI events) and individual components of the Framingham Risk Score (sex, age, systolic BP, total cholesterol, HDL, diabetes, smoking) (secondary analysis).

**Table 1.** Sample Characteristics

Variable	Whole sample, No. (%) (N = 617)	Group differences		
		No TBI (n = 394)	With TBI (n = 223)	*P* value
Sex				
Female	380 (61.6)	273 (69.3)	107 (48.0%)	<.001
Male	237 (38.4)	121 (30.7)	116 (52.0%)	
Age, mean (SD), y	51.2 (5.5)	51.4 (5.4)	50.8 (5.6)	.19
Education, mean (SD), y	16.7 (3.5)	16.9 (3.6)	16.5 (3.2)	.16
APOE4	233 (37.8)	153 (38.8)	80 (35.9)	.49
Family history	323 (52.4)	213 (54.1)	110 (49.3)	.30
Hypertension	106 (17.2)	59 (15.0)	47 (21.1)	.07
Hyperlipidaemia	74 (12.0)	45 (11.4)	29 (13.0)	.70
Diabetes	17 (2.8)	11 (2.8)	6 (2.7)	>.99
BMI, mean (SD)	27.4 (5.1)	27.4 (5.1)	27.5 (5.1)	.79
Current smoker	35 (5.7)	15 (3.8)	20 (9.0)	.01
High alcohol intake^a^	88 (14.3)	52 (13.2)	36 (16.1)	.38
CVD risk, mean (SD)^b^	8.0 (3.9)	7.8 (3.7)	8.4 (4.2)	.12

Abbreviations: APOE4, apolipoprotein E ε4; BMI, body mass index (calculated as weight in kilograms divided by height in meters squared); CVD risk, cardiovascular disease risk; TBI, traumatic brain injury.

^a^ High alcohol intake is defined as more than 21 units per week.

^b^ CVD risk assessed using the Framingham Risk Score.

All analyses were conducted in the full sample and repeated in a subgroup of individuals to compare mild TBI and non-TBI controls (eAppendix in Supplement 1), where applicable. As part of sensitivity analysis, TBI-SVD associations and moderation analyses were repeated to include the additional covariates of (1) smoking and (2) smoking, APOE4, hypertension, hyperlipidaemia, diabetes, alcohol, and Framingham Risk Score for CVD. All analyses were conducted using *R* version 4.4.0 (R Project for Statistical Computing) with statistical significance set at *P* = .05 in 2-sided tests.

## Results

### Cohort Characteristics

Of the 617 participants (median [IQR] age, 52 [47-56] years; 380 women [51.6%]; median [IQR] education, 17 [14-19] years), approximately one-third of participants (223 [36.1%]) reported at least 1 TBI with LOC; of these, 125 (56.1%) reported a single event, 61 (27.4%) reported 2 TBI events, and 37 (16.6%) reported more than 2 events (Table 1). Of the 223 participants with a history of TBI, 170 (76.2%) had sufficient information reported on the BISQ to determine injury severity, of which 160 (94.1%) reported a history of mTBI and 10 (5.9%) moderate-severe incidents (LOC 30 minutes or longer). TBI was more common in male (116 [48.9%]) than female participants (107 [28.2%]) (OR, 2.45; 95% CI, 1.74-3.43), but did not differ by age, education, APOE4, hypertension, hyperlipidaemia, diabetes, alcohol intake or CVD risk score (Table 1). However, individuals with prior TBI were more likely to be current smokers (OR, 2.48; 95% CI, 1.24-4.94), even after controlling for sex (OR, 2.28; 95% CI, 1.13-4.70). CMB were detected in approximately 1 in 6 participants (109 [17.7%]); most had 1 to 3 CMB recorded (103 [16.7%]), while very few had more than 3 CMB (6 [1.0%]). Regionally, lobar CMB were detected in 86 participants (13.9%), deep CMB in 30 (4.9%), and infratentorial CMB in 2 (0.3%); 9 participants (1.5%) had both deep and lobar CMB.

### Association of SVD Markers With CVD Risk Score

As expected, higher CMB counts were associated with older age (β = 0.07; 95% CI, 0.03-0.11), adjusting for sex, education, and study site; those aged 50 years or more were 1.81 (β = 0.05; 95% CI, 1.14-2.95) times more likely to have CMB. However, CMB were not related to CVD risk, contrary to other SVD markers which were positively associated with CVD risk. Specifically, higher CVD risk was associated with greater WMH volume, lacunes, and PVS (in BG, but not CSO) (eTable 2 in Supplement 1).

### Association of TBI With SVD Markers

In adjusted analysis, CMB numbers were greater in individuals with prior TBI than in those without TBI, with a dose-response association observed with increasing number of TBI events (95% CI, 0.01-0.09) (eTable 3 in Supplement 1). By contrast, TBI history was not associated with WMH volume, lacunes, or PVS. This association held for mTBI, where CMB, but not other SVD markers, were associated with mTBI history. Results were unchanged in sensitivity analyses adjusting for the additional covariate(s) of smoking alone (model 2), or smoking, APOE4, hypertension, hyperlipidaemia, diabetes, alcohol, and CVD risk score (model 3) (eTable 3 in Supplement 1).

### Moderation Analysis of TBI on the Association Between CMB and Vascular Risk Factors

**Figure 1.**
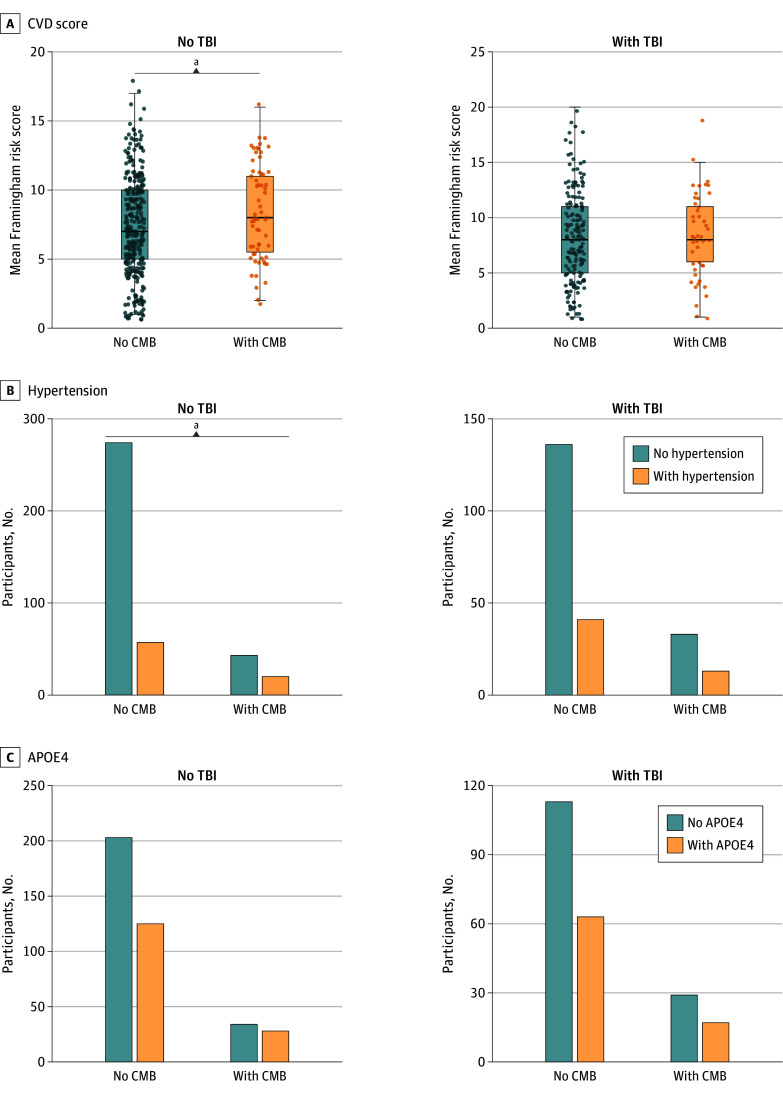
Cerebral Microbleeds Associated With Modifiable Vascular Risk Factors in the Absence of TBI History Interaction analyses were conducted using general linear regression models fitted to each variable of interest in separate models, adjusting for sex, age, education, and study site. In panel A, dots indicate Framingham Risk Score results for individual participants. APOE4 indicates apolipoprotein E ε4; CMB, cerebral microbleeds; CVD, cardiovascular disease risk; TBI, traumatic brain injury. ^a^*P* < .05.

In interaction analyses adjusting for age, sex, education, and study site, TBI × CMB interaction on CVD risk score was significant. Specifically, the association between CMB and higher CVD risk was diminished as number of TBI events increased; regionally, this was observed for lobar CMB, but not global or deep CMB. This was also observed when analyzing hypertension alone, where lobar and global CMB interactions with TBI on CVD were present, but not deep CMB. There was no TBI × CMB interaction in relation to APOE4 (Figure 1, Table 2).

**Table 2.** Moderating Effect of Traumatic Brain Injury (TBI) on the Association Between Cerebral Microbleeds (CMB) and Clinical Measures

Outcome	T value	*P* value	Estimate, β (95% CI)
**Clinical measures^a^**			
Memory			
Global CMB^b^	2.35	.02	0.14 (0.02 to 0.26)
Lobar CMB^b^	2.03	.04	0.14 (0.004 to 0.27)
Deep CMB	1.06	.29	0.11 (−0.09 to 0.31)
Language			
Global CMB^b^	2.07	.04	0.14 (0.01 to 0.27)
Lobar CMB^b^	2.10	.04	0.16 (0.01 to 0.30)
Deep CMB	−0.62	.54	−0.07 (−0.30 to 0.15)
Attention			
Global CMB	1.72	.09	0.09 (−0.01 to 0.20)
Lobar CMB	1.59	.11	0.09 (−0.02 to 0.21)
Deep CMB	0.80	.43	0.07 (−0.11 to 0.26)
Visuospatial			
Global CMB	−0.76	.45	−0.05 (−0.17 to 0.07)
Lobar CMB	0.09	.93	−0.01 (−0.14 to 0.13)
Deep CMB	−0.76	.45	−0.08 (−0.28 to 0.13)
Gait			
Global CMB^b^	−2.19	.03	−0.03 (−0.06 to −0.004)
Lobar CMB	−0.89	.38	−0.02 (−0.05 to 0.02)
Deep CMB^b^	−2.65	.01	−0.07 (−0.12 to −0.02)
**Risk factors** ^a^			
CVD risk score^c^			
Global CMB	−1.70	.09	−0.06 (−0.13 to 0.01)
Lobar CMB^b^	−2.61	.01	−0.10 (−0.18 to −0.02)
Deep CMB	0.90	.37	0.06 (−0.07 to 0.18)
Hypertension			
Global CMB	−1.37	.17	−0.35 (−0.86 to 0.15)
Lobar CMB^b^	−2.38	.02	−0.73 (−1.32 to −0.14)
Deep CMB	0.54	.59	0.22 (−0.60 to 1.06)
APOE4			
Global CMB	−0.39	.70	−0.09 (−0.52 to 0.34)
Lobar CMB	0.94	.35	0.23 (−0.26 to 0.73)
Deep CMB	−1.13	.26	−0.43 (−1.23 to 0.30)

Abbreviations: APOE4, apolipoprotein E ε4; CMB, cerebral microbleeds; CVD, cardiovascular disease.

^a^ Analyses were adjusted for sex, age, years of education, and study site. General linear regression models were fitted for the continuous outcome variables of memory, language, and CVD risk score (estimates and 95% CI are in standardized units), while logistic regression was applied for the binary outcome variables of gait, hypertension, and APOE4 (estimates and 95% CI are in log-odds).

^b^ Statistically significant; *P* < .05, 95% CI does not include zero.

^c^ Framingham Risk Score.

### Moderation Analysis of TBI on Associations Between CMB and Clinical Outcome Measures

**Figure 2.**
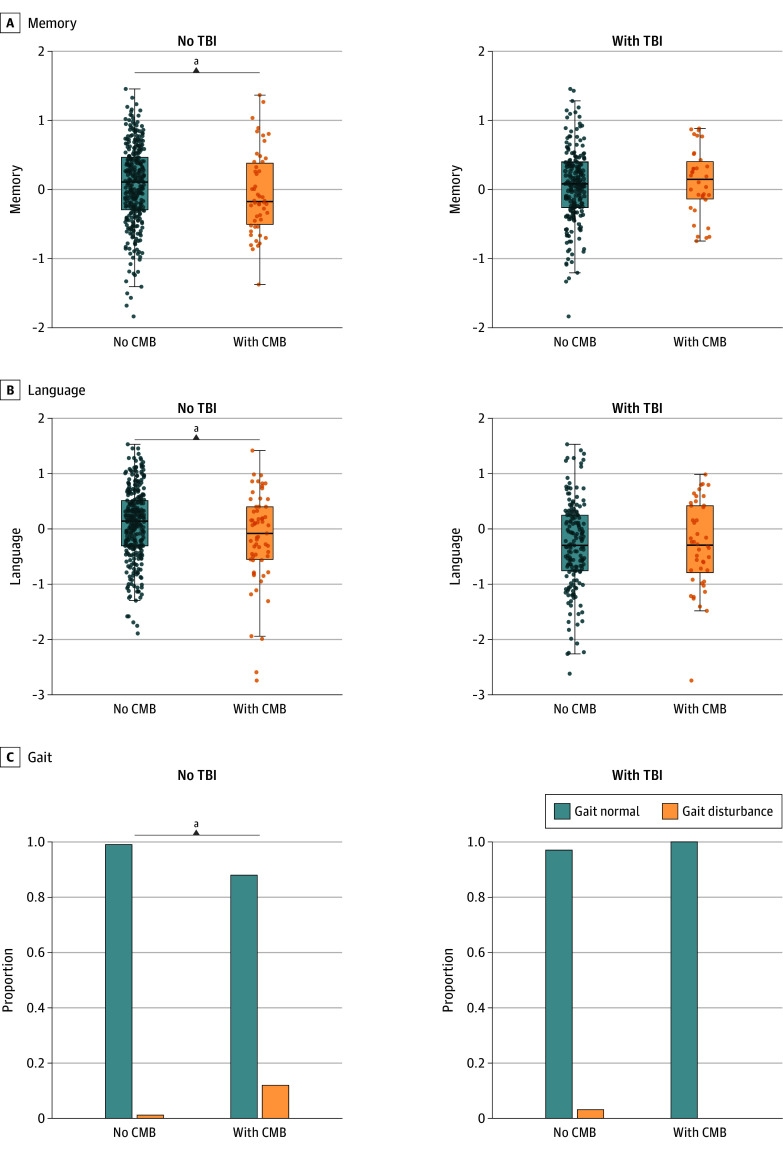
Cerebral Microbleeds Associated With Poorer Clinical Outcomes in the Absence of TBI History Interaction analyses were conducted using linear regression models fitted to each variable of interest in separate models, adjusting for sex, age, education, and study site. In panels A and B, dots represent individual measures of clinical outcomes. CMB indicates cerebral microbleeds; TBI, traumatic brain injury. ^a^*P* < .05.

Significant TBI × CMB interactions on cognition were demonstrated for the domains of memory and language, whereby associations between CMB and cognition were diminished as number of TBI events increased (Figure 2; Table 2). Again, these applied to lobar, but not deep, CMB. No TBI × CMB interactions were detected on attention or visuospatial function. Similar interactions were observed in relation to gait, although regional patterns were reversed, whereby TBI interacted with deep, but not lobar, CMB. Again, observations held in mTBI subgroup analyses, such that mTBI × CMB interactions were demonstrated in relation to memory and gait but not attention or visuospatial function.

### Association of TBI With Clinical Deficits

In adjusted regression models, greater numbers of TBI events were associated with poorer sleep, gait disturbances, greater depression symptoms, and memory but not attentional deficits (eTable 4 in Supplement 1). These findings were echoed in the mTBI subgroup, where higher numbers of TBI events were related to poorer sleep, depression, and gait, but not any cognitive domain. Results were unchanged in sensitivity analysis adjusting for different sets of covariates (eTable 4 in Supplement 1).

**Figure 3.**
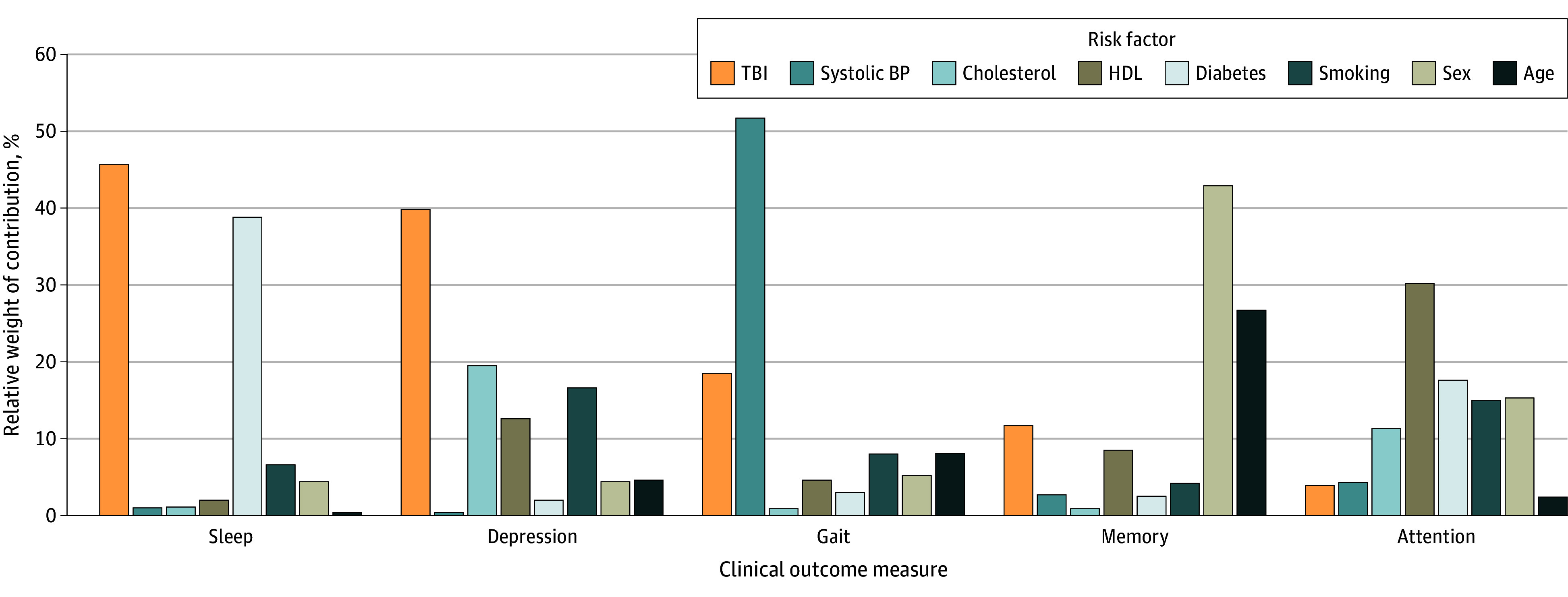
Relative Contribution of Risk Factors on Clinical Outcome Measures Bar heights represent standardized general dominance statistics, which may be interpreted as the relative weight of contribution to *R^2^*. BP indicates blood pressure; HDL, high-density lipoprotein; TBI, traumatic brain injury.

Within the TBI group, we examined the relative contribution of TBI and CVD risk to clinical deficits using dominance analysis and relative weights analysis. Results demonstrated that TBI itself was the most important factor contributing toward depression and sleep (but not cognition or gait), outweighing the contribution of individual CVD risk factors (Figure 3). In relation to depression, TBI achieved complete dominance over each individual CVD exposure variables (contribution to *R^2^* = 38.5%). In relation to sleep, TBI displayed complete dominance over all CVD variables except diabetes, which TBI generally (but not completely) dominated (TBI, 47.1% vs diabetes, 36.2%). In relation to gait disturbances, TBI had complete dominance over all other exposure variables except for systolic BP, which was in complete dominance of TBI and all other factors (TBI, 17.7% vs systolic BP, 51.0%). While TBI dominated CVD risk factors (eg, diabetes, high BP) in contributing toward memory deficits, the main dominating factors were sex and age. On the other hand, TBI was not a key contributor to attentional deficits.

## Discussion

These data demonstrate that in otherwise healthy middle-aged adults, remote TBI history was associated with detectable changes in vascular brain imaging and clinical features. Among the SVD markers analyzed, MRI-detected cerebral microbleeds (CMB) were the only markers (1) associated with TBI and (2) not to the Framingham Risk Score. Conversely, all other SVD markers were associated with CVD risk but not TBI. Intriguingly, CMB associations with cardiovascular disease (CVD) risk emerged only in the subset of individuals without prior TBI history, but not in the TBI subgroup. CMB associations with various clinical measures were also moderated by TBI, such that effect sizes in the association between CMB and clinical deficits were diminished in those with TBI. Importantly, findings were replicated in analysis restricted to mild TBI.

The lack of association between CMB and vascular risk factors in the TBI group suggests that CMB in these individuals may be less likely driven by chronic vascular pathology, and more likely to be acquired from TBI (what may be described as traumatic CMB). In support of this, results were regionally restricted to lobar CMB but not deep CMB. This is consistent with the observation that while wider chronic vascular disease more typically relates to the development of subcortical CMB,^44,45^ lobar CMB often stem from origins other than underlying vascular disease, including TBI.^4,5^ While further research on the regional distribution of traumatic CMB is required, findings suggest that CMB detected in lobar regions warrant greater consideration of nonvascular causes, especially when detected in younger individuals.

In the non-TBI group, CMB burden was associated with the expected cognitive and functional impairments characteristic of SVD, although these associations were diminished in those with TBI history. The greater clinical impairments accompanying CMB in our non-TBI group could be attributed to underlying chronic vascular alterations and more widespread cerebrovascular dysfunction. That said, longitudinal analyses of larger prospective cohorts will be needed to (1) elucidate longer-term effects of traumatic CMB, which did not relate to clinical impairments at baseline but may confer differential effects on long-term clinical trajectory and/or downstream pathological processes compared with vascular CMB, and (2) shed light on the mechanistic link between TBI and CMB, by examining whether TBI-related CMB occur as a direct result of the impact (eg, shear forces) or by initiating microvascular fragility leading to higher risk of developing CMB.^5^

Sex differences were consistent with past research, whereby TBI were more prevalent in men than in women.^46^ Within our cohort of healthy middle-aged adults, almost half of male participants (48.7%) reported at least 1 TBI event, compared with 28.1% of female participants. While male participants tend to present with greater SVD burden compared with female participants,^47^ we previously found that this sex difference was rendered nonsignificant once accounting for modifiable dementia risk factors (including TBI),^28^ implying that sex differences in SVD could be attributed to greater prevalence of certain risk factors in men.^48^ Taken together with our present findings, the cumulative evidence underscores the importance of considering sex differences to elucidate the pathophysiology and etiology of CMB, and perhaps SVD by extension.

Overall, our findings have important implications for future research directions, as well as informing clinical practices and policymaking at the community level. While CMB are largely presumed to originate from chronic vascular etiology, our results draw attention to alternative explanations such as TBI. Using a structured TBI screening tool, we identified a sizable proportion of individuals with TBI history within a healthy middle-aged community sample. Therefore, trauma-induced CMB are likely to be present in most research samples and may distort results and interpretations of analyses that are performed on the assumption that CMB represents chronic SVD. When unaccounted for, TBI may obscure the clinical importance of CMB, potentially explaining the inconsistent results in prior research regarding CMB associations with clinical correlates that are well-established in other SVD markers.

Clinically, recognizing the differential etiologies and clinical consequences between traumatic vs vascular CMB could help clinicians by informing treatment decisions and improving prognostic accuracy. While the primary insult of the TBI event cannot be reversed, appropriate clinical interventions could mitigate the downstream cascade of secondary damage to the cerebrovascular network, eg, cerebral blood flow, inflammation, blood-brain barrier, and oxidative stress.^22^ Therefore, we recommend the inclusion of TBI assessments in relevant research studies and clinical assessments to aid the interpretation of underlying etiologies and their clinical implications.

In the community, interventions could focus on prevention (eg, improving safety in sports and recreation and occupational health safety standards) and public education campaigns (eg, raising awareness of the activities associated with TBI risk, recognition of symptoms and encouraging prompt medical attention following TBI). Therefore, while risk modification often focuses on the role of individuals to modify their own behaviors, structural interventions (eg, through policymaking) will be fundamental to reducing risk at a societal level, and more equitably.^49^

### Limitations

Study limitations include potentially limited generalizability of findings to the broader population given the predominantly White and higher-educated participants in our cohort.^29^ Furthermore, self-reported measures based on retrospective recall are susceptible to misreporting, although this is mitigated through the structured BISQ tool, which prompts recall of specific situations where head injuries could occur. For CMB detection, future studies with greater CMB burden could benefit from automated methods (eAppendix in Supplement 1). Other fundamental limitations include volunteer bias and the cross-sectional design.

## Conclusions

History of TBI, even mild TBI, was associated with detectable alterations in clinical and neuroimaging measures as early as midlife. These findings highlight the potential brain health consequences of TBI and provide one possible pathological mechanism driving TBI-related neurodegeneration. Our data suggest that TBI-related clinical features (eg, depression, poorer sleep) may operate via cerebrovascular injury, which also correlate with other dementia risk factors. These observations underscore potential intervention targets for dementia prevention in patients with TBI, and avenues for future research.

## Notes

Supplement 1. **eFigure.** Participant Selection Flowchart **eAppendix.** Supplementary Methods **eTable 1.** Sample Characteristics of Subset of mTBI Comparison Groups **eTable 2.** Association Between Cardiovascular Disease (CVD) Risk and Cerebral Small Vessel Disease (SVD) **eTable 3.** Association Between Traumatic Brain Injury (TBI) and Cerebral Small Vessel Disease (SVD) **eTable 4.** Association Between Traumatic Brain Injury (TBI) and Clinical Features

Supplement 2.
Nonauthor Collaborators

Supplement 3.
Data Sharing Statement
