# Supplementary material for: Neuroimaging and Clinical Findings in Healthy Middle-Aged Adults With Mild Traumatic Brain Injury in the PREVENT Dementia Study
Source: JAMA Netw Open. 2024 Aug 15;7(8):e2426774. doi: 10.1001/jamanetworkopen.2024.26774 (PMC11327885; doi:10.1001/jamanetworkopen.2024.26774)
Supplement: Supplement 1. — eFigure. Participant Selection Flowchart eAppendix. Supplementary Methods eTable 1. Sample Characteristics of Subset of mTBI Comparison Groups eTable 2. Association Between Cardiovascular Disease (CVD) Risk and Cerebral Small Vessel Disease (SVD) eTable 3. Association Between Traumatic Brain Injury (TBI) and Cerebral Small Vessel Disease (SVD) eTable 4. Association Between Traumatic Brain Injury (TBI) and Clinical Features [file jamanetwopen-e2426774-s001.pdf]

Supplemental Online Content

Low A, McKiernan E, Prats-Sedano MA, et al; and the PREVENT Dementia Investigators. Neuroimaging and Clinical Findings in Healthy Middle-Aged Adults With Mild Traumatic Brain Injury. *JAMA Netw Open.* 2024;7(8):e2426774. doi:10.1001/jamanetworkopen.2024.26774

eFigure. Participant Selection Flowchart

eAppendix. Supplementary Methods

eTable 1. Sample Characteristics of Subset of mTBI Comparison Groups

eTable 2. Association Between Cardiovascular Disease (CVD) Risk and Cerebral Small Vessel Disease (SVD)

eTable 3. Association Between Traumatic Brain Injury (TBI) and Cerebral Small Vessel Disease (SVD)

eTable 4. Association Between Traumatic Brain Injury (TBI) and Clinical Features

This supplemental material has been provided by the authors to give readers additional information about their work.

© 2024 Low A et al. *JAMA Network Open.*

eFigure 1. Participant selection flowchart.

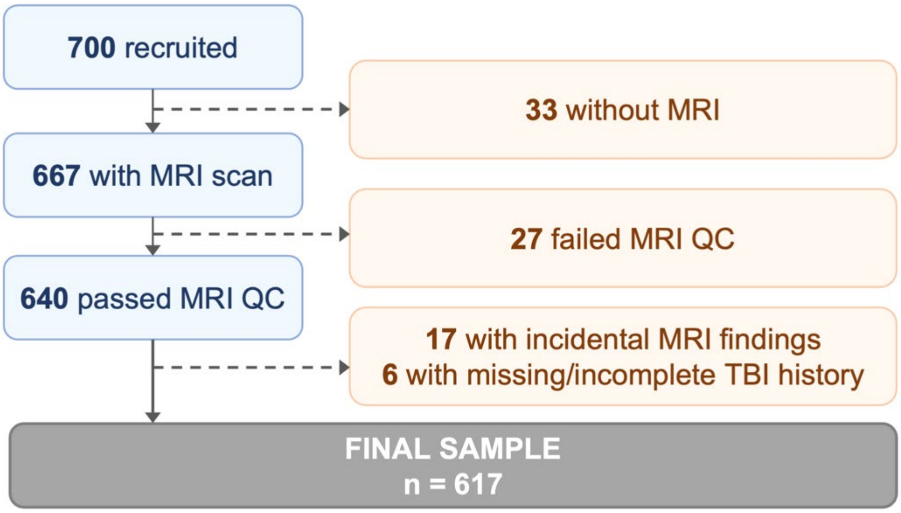

© 2024 Low A et al. *JAMA Network Open.*

eAppendix. Supplementary Methods.

Participant recruitment

Cognitively healthy individuals, aged 40 to 59, were recruited to the PREVENT Dementia programme through various channels. Initially, potential participants were sourced from healthy controls registered with the dementia register database maintained by the West London Mental Health National Health Service (NHS) Trust. As well as people with cognitive impairment and dementia, the database contains information on health individuals (often caregivers of people with dementia or their unaffected children) who have given consent to be approached for clinical research. Additionally, the study sought to recruit healthy control individuals registered on the Join Dementia Research website or those who expressed interest through the PREVENT-Dementia website, as well as attendees of public presentations and engagement sessions, and through word of mouth. Further details on the study design, recruitment procedures, and sample composition have been previously published.[1,2] Potential participants were screened for eligibility. Participants had to be aged 40-59, have no known contraindications to having an MRI scan and could not have a diagnosis or be under assessment for any form of cognitive impairment or dementia. Individuals deemed eligible were provided with a Participant Information Sheet, and a trained member of the study team introduced the project and obtained informed consent. The PREVENT Dementia programme received multi-site ethical approval from the UK London-Camberwell St Giles NHS Research Ethics Committee (REC reference: 12/LO/1023, IRAS project ID: 88938), which operates according to the Helsinki Declaration of 1975 (and as revised in 1983). A separate ethical application was submitted for the Dublin site in Ireland, which was reviewed and given a favourable opinion by Trinity College Dublin School of Psychology Research Ethics Committee (SPREC022021-010) and the St James Hospital/Tallaght University Hospital Joint Research Ethics Committee. All substantial protocol amendments have been reviewed by the same ethics committees and favourable opinion was granted before implementation .

Clinical assessments

Blood pressure was measured in triplicate after five minutes of supine rest. Hypertension, hyperlipidaemia, and diabetes mellitus were analysed as binary variables. The presence of hypertension was defined as having an average diastolic blood pressure ≥140 mmHg, systolic blood pressure ≥90 mmHg, or a positive report of hypertension in their medical history interview. The presence of hyperlipidaemia was defined as total cholesterol >6.5 mmol/L or a positive report of hyperlipidaemia in their medical history interview. The presence of diabetes mellitus was defined as glucose level ≥7 mmol/L or a positive report of diabetes mellitus in their medical history interview. Physical and neurological examinations were completed by a qualified doctor, e.g., gait. Alcohol intake and smoking status were assessed through a lifestyle interview. Participants were first asked if they were a non-smoker/drinker, ex-smoker/drinker, or current smoker/drinker. Ex-/current drinkers were asked to estimate the number of glasses of wine, beer, and stronger alcohol (e.g., hard liquor) consumed per week, and the total number of units per day/week was computed. 'High' alcohol intake was binarized at a cut-off of >21 units per week.

MRI acquisition parameters

Three-dimensional T1-weighted MPRAGE parameters: 160 slices, repetition time (TR)=2300ms, echo time (TE)=2.98ms, flip angle=9°, voxel size=1 × 1 × 1mm^3. T2-weighted parameters: 32 slices, TR=1500ms, TE=80ms, flip angle=150°, voxel size=0.69 × 0.69 × 4mm^3. SWI MRI parameters: 72 slices, TR=28ms, TE=20ms, flip angle=15°, voxel size=0.72 × 0.72 × 1.2mm^3. Fluid-attenuated inversion recovery (FLAIR) parameters: 27 slices, TR=9000ms, TE=94ms, flip angle=150°, voxel size=0.43 × 0.43 × 4mm^3.

Quantification of cerebral small vessel disease (SVD)

For CMB detection, improved sensitivity was afforded by the acquisition of thin-sliced (vs. thick-sliced) 3T (vs. 1.5T) SWI (vs. conventional gradient echo sequences; GRE) scans, which are reported to detect up to triple the number of CMB compared to conventional 1.5T GRE scans,[6] and are thought to represent the true prevalence of CMB with greater accuracy and reliability. Suspected CMB were cross-validated on T1- and T2-weighted images to exclude CMB 'mimics' (e.g., melatonic melanoma). Where uncertain, CMB were labelled as 'possible CMB' – this includes scenarios whereby CMB cannot be distinguished from vascular flow voids. Such cases of 'possible CMB' were excluded from analysis, and only 'definite CMB' were analysed. Lacunes and CMB were classified by location as deep or lobar. Lobar regions were defined according to Stark and Bradley,[7] comprising cortical and subcortical regions, while deep regions included the basal ganglia, thalamus, internal capsule, external capsule, corpus callosum, and deep and periventricular white matter.[8,9] Visual ratings of each SVD marker were done by a single rater, and 20% of scans were rated by a second rater. The subset of 20% was derived from a random

© 2024 Low A et al. *JAMA Network Open.*

sampling of all participants stratified by study site, performed by a blinded third party who was not involved in any SVD ratings. Raters were blinded to all clinical information, and inter-rater reliability results (Cohen's kappa) were as follows: CMB: 0.74, lacunes: 0.92, PVS: 0.90 in centrum semiovale, 0.85 in basal ganglia, WMH: 0.74 for periventricular, and 0.89 for deep.[5]

Neuropsychological assessments and questionnaires

The COGNITO battery comprises test components that are well-established to have high discriminability in the detection of underlying brain pathology and has demonstrated validity in a range of clinical phenotypes, and test-retest reliability for most sub-tests.[1,11–13] Composite cognitive domain scores were computed by averaging the z-scores of relevant tasks in each cognitive domain using cohort-derived means and standard deviations. The composite *memory* score was derived from recall tasks in the COGNITO battery, including tasks of name recall, face recall, narrative recall, name-face association, and a test of implicit memory. The composite score for *language* included measures of sentence comprehension, verbal fluency, and vocabulary. The composite *attention* score was made up of visual attention, auditory attention, visual-auditory attention, and reaction time. Further details on the COGNITO have been previously described.[1,14]

The Pittsburgh Sleep Quality Index (PSQI),[15] comprises 19 items to assess sleep duration, sleep disturbances, etc. The PSQI score ranges from 0 to 21, whereby higher scores indicate poorer sleep quality. The PSQI is one of the most commonly used measures of sleep quality and has been validated widely, demonstrating good test-retest correlation across its seven components,[16] internal consistency reliability, and construct validity (both convergent and discriminant validity).[17]
Depression was assessed using the Center for Epidemiologic Studies Depression Scale (CES-D).[18] The CES-D is a 20-item self-report scale designed to assess symptoms of depression, with scores ranging from 0 to 60, such that higher scores indicate greater depressive symptoms. The CES-D is established to have broad applicability to the general population and has been validated in both healthy populations and patient groups, and across age groups and cultures, demonstrating high internal consistency and good test-retest reliability.[18,19]

Assessment of traumatic brain injury

History of TBI was assessed using the Brain Injury Screening Questionnaire (BISQ),[20] which was designed to prompt recall of past injuries by including questions about specific situations where head injuries could occur. This structured methodology is considered the gold standard for the screening of TBI.[21] In contrast, single-item methods (e.g., "Have you ever had a head injury?") tend to underestimate TBI prevalence, missing more than one-third of individuals who were found to have experienced TBI through a subsequent structured interview.[22,23] The BISQ has demonstrated good validity and sensitivity across a range of settings and populations including schoolchildren, adults, and high-risk populations, showing good construct validity and criterion validity, and has been observed to be more sensitive in detecting TBI history when compared to other measures.[20,24] While informant data are not collected in our PREVENT cohort, the BISQ has demonstrated good agreement between participant and informant scores.[25]

To further examine whether results extended to *mild* TBI (mTBI), we composed a separate grouping variable where mTBI+ was defined as a subset of the TBI+ group whose TBI-related unconsciousness did not exceed 30 minutes, as per the American Congress of Rehabilitation Medicine (ACRM) diagnostic criteria for mild TBI.[26] 564 participants had sufficient information reported on the BISQ to determine *mild* TBI status (i.e., presence of head injury with loss of consciousness, and if present, duration of period of unconsciousness), and 10 were excluded from mTBI group comparisons as they reported periods of unconsciousness of 30 minutes or more, i.e., moderate-severe TBI, leaving a total of n=554 participants for mild TBI group comparisons.

Statistical analysis

Normality of continuous data was tested using the Shapiro–Wilk test. Parametric data were analysed using independent *t*-tests or analysis of variance (ANOVA), and non-parametric data were analysed using the Wilcoxon rank-sum test or Kruskal-Wallis test. Chi-square tests of independence were conducted for group comparisons of categorical variables. Cube-root transformation was applied to variables with skewed distributions, including the normalised WMH volume, number of CMB, number of lacunes, number of TBI events, CVD risk score. Prior to the computation of Framingham Risk Scores, missing data were imputed using the *mice* package in R to perform multiple imputation – data on diabetes and smoking were missing for n=2 (0.3%), while cholesterol data were missing for n=17 (2.8%). Little's test determined that data was *missing completely at random* (MCAR; p=0.10).

© 2024 Low A et al. *JAMA Network Open.*

To compare the relative contribution of TBI and vascular risk factors to clinical deficits in the subset of TBI+ participants, we conducted dominance analysis and relative weights analysis using the *domir* package in R.

Dominance analysis is used to evaluate the relative importance of predictor variables in explaining the variance in the outcome variable by examining the change in R^2 that occurs when each individual predictor is added to all possible permutations of subsetted regression models.[27] Because this method accounts for interactions and collinearity between predictors, dominance analysis is preferred over traditional tests of relative importance (e.g., standardized regression coefficients, squared zero-order correlations, squared beta weights).[28,29] Dominance analysis produces a dominance matrix comparing every predictor pair to evaluate whether one predictor is dominant over another based on general dominance statistics, conditional dominance statistics, and complete dominance statistics. General dominance is simply achieved if the averaged additional contribution (R^2) of one predictor exceeds the other and represent the Shapley value decompositions of the fit statistic. Conditional dominance is similar but more stringent, and is achieved when a predictor's average additional contribution within each model size is greater than that of another predictor. The final and most stringent form of dominance is complete dominance, which is achieved if the averaged additional contribution of one predictor exceeds that of another predictor across all possible models. Complete dominance implies that both general and conditional dominance have been established, though the reverse cannot be assumed.[29]

© 2024 Low A et al. *JAMA Network Open.*

eReferences

1. Ritchie K, de Roquefeuil G, Ritchie CW, et al. COGNITO: Computerized Assessment of Information Processing. *J Psychol Psychother*. 2014;4(2):136. doi:10.4172/2161-0487.1000136
2. Ritchie CW, Ritchie K. The PREVENT study: A prospective cohort study to identify mid-life biomarkers of late-onset Alzheimer's disease. *BMJ Open*. 2012;2(6):1-6. doi:10.1136/bmjopen-2012-001893
3. Low A, Prats-Sedano MA, Stefaniak JD, et al. CAIDE dementia risk score relates to severity and progression of cerebral small vessel disease in healthy midlife adults: the PREVENT-Dementia study. *J Neurol Neurosurg Psychiatry*. 2022;93(5):481-490. doi:10.1136/jnnp-2021-327462
4. Low A, Su L, Stefaniak JD, et al. Inherited risk of dementia and the progression of cerebral small vessel disease and inflammatory markers in cognitively healthy midlife adults: the PREVENT-Dementia study. *Neurobiol Aging*. 2021;98:124-133. doi:10.1016/j.neurobiolaging.2020.10.029
5. Low A, Prats-Sedano MA, McKiernan E, et al. Modifiable and non-modifiable risk factors of dementia on midlife cerebral small vessel disease in cognitively healthy middle-aged adults: the PREVENT-Dementia study. *Alzheimers Res Ther*. 2022;14(1):1-13. doi:10.1186/S13195-022-01095-4
6. Nandigam RNK, Viswanathan A, Delgado P, et al. MR imaging detection of cerebral microbleeds: Effect of susceptibility-weighted imaging, section thickness, and field strength. *American Journal of Neuroradiology*. 2009;30(2):338-343. doi:10.3174/ajnr.A1355
7. Stark DD, Bradley Jr WG. *Magnetic Resonance Imaging*. Vol 19922. 3rd ed.; 1999.
8. Gregoire SM, Chaudhary UJ, Brown MM, et al. The Microbleed Anatomical Rating Scale (MARS): reliability of a tool to map brain microbleeds. *Neurology*. 2009;73(21):1759-1766. doi:10.1212/WNL.0b013e3181c34a7d
9. Pasi M, Boulouis G, Fotiadis P, et al. Distribution of lacunes in cerebral amyloid angiopathy and hypertensive small vessel disease. *Neurology*. 2017;88(23):2162-2168. doi:10.1212/WNL.0000000000004007
10. Potter GM, Chappell FM, Morris Z, Wardlaw JM. Cerebral perivascular spaces visible on magnetic resonance imaging: development of a qualitative rating scale and its observer reliability. *Cerebrovasc Dis*. 2015;39(4):224-231. doi:10.1159/000375153
11. Ancelin ML, De Roquefeuil G, Ledésert B, Bonnel F, Cheminal JC, Ritchie K. Exposure to anaesthetic agents, cognitive functioning and depressive symptomatology in the elderly. *The British Journal of Psychiatry*. 2001;178(4):360-366. doi:10.1192/BJP.178.4.360
12. Ritchie K, Artero S, Touchon J. Classification criteria for mild cognitive impairment: A population-based validation study. *Neurology*. 2001;56(1):37-42. doi:10.1212/WNL.56.1.37
13. Ancelin ML, De Roquefeuil G, Scali J, et al. Long-Term Post-Operative Cognitive Decline in the Elderly: The Effects of Anesthesia Type, Apolipoprotein E Genotype, and Clinical Antecedents. *Journal of Alzheimer's Disease*. 2010;22(s3):S105-S113. doi:10.3233/JAD-2010-100807
14. Ritchie K, Carrière I, Su L, et al. The midlife cognitive profiles of adults at high risk of late-onset Alzheimer's disease: The PREVENT study. *Alzheimer's and Dementia*. 2017;13(10):1089-1097. doi:10.1016/j.jalz.2017.02.008
15. Buysse DJ, Reynolds CF, Monk TH, Berman SR, Kupfer DJ. The Pittsburgh sleep quality index: A new instrument for psychiatric practice and research. *Psychiatry Res*. 1989;28(2):193-213. doi:10.1016/0165-1781(89)90047-4
16. Sohn S Il, Kim DH, Lee MY, Cho YW. The reliability and validity of the Korean version of the Pittsburgh Sleep Quality Index. *Sleep and Breathing*. 2012;16(3):803-812. doi:10.1007/S11325-011-0579-9
17. Carpenter JS, Andrykowski MA. Psychometric evaluation of the Pittsburgh Sleep Quality Index. *J Psychosom Res*. 1998;45(1):5-13. doi:10.1016/s0022-3999(97)00298-5
18. Radloff LS. The CES-D Scale: A Self-Report Depression Scale for Research in the General Population. *Appl Psychol Meas*. 1977;1(3):385-401. doi:10.1177/014662167700100306
19. Jiang L, Wang Y, Zhang Y, et al. The reliability and validity of the Center for epidemiologic Studies Depression Scale (CES-D) for Chinese university students. *Front Psychiatry*. 2019;10(315):456760. doi:10.3389/FPSYT.2019.00315
20. Dams-O'Connor K, Cantor JB, Brown M, Dijkers MP, Spielman LA, Gordon WA. Screening for traumatic brain injury: findings and public health implications. *J Head Trauma Rehabil*. 2014;29(6):479-489. doi:10.1097/HTR.0000000000000099
21. Corrigan JD, Bogner J. Screening and identification of TBI. *Journal of Head Trauma Rehabilitation*. 2007;22(6):315-317. doi:10.1097/01.HTR.0000300226.67748.3E
22. Diamond PM, Harzke AJ, Magaletta PR, Cummins AG, Frankowski R. Screening for traumatic brain injury in an offender sample: A first look at the reliability and validity of the traumatic brain injury questionnaire. *Journal of Head Trauma Rehabilitation*. 2007;22(6):330-338. doi:10.1097/01.HTR.0000300228.05867.5C

© 2024 Low A et al. *JAMA Network Open.*

23. Russell LM, Devore MD, Barnes SM, et al. Challenges Associated With Screening for Traumatic Brain Injury Among US Veterans Seeking Homeless Services. *Am J Public Health*. 2013;103(Suppl 2):S211-S212. doi:10.2105/AJPH.2013.301485
24. Makri A, Koulenti A, Argyrou K, Gordon W, Constantinidou F. The Risk for Traumatic Brain Injury and Persisting Symptomatology in Elementary, Secondary, and University-Level Students: An International Perspective With the Greek Version of the Brain Injury Screening Questionnaire. *Top Lang Disord*. 2019;39(3).
25. Cantor JB, Gordon WA, Schwartz ME, Charatz HJ, Ashman TA, Abramowitz S. Child and parent responses to a brain injury screening questionnaire. *Arch Phys Med Rehabil*. 2004;85(4 Suppl 2):S54-60. doi:10.1016/j.apmr.2003.08.113
26. Silverberg ND, Iverson GL, Cogan A, et al. The American Congress of Rehabilitation Medicine Diagnostic Criteria for Mild Traumatic Brain Injury. *Arch Phys Med Rehabil*. 2023;104(8):1343-1355. doi:10.1016/j.apmr.2023.03.036
27. Tonidandel S, LeBreton JM. Relative Importance Analysis: A Useful Supplement to Regression Analysis. *J Bus Psychol*. 2011;26(1):1-9. doi:10.1007/S10869-010-9204-3
28. Johnson JW, Lebreton JM. History and Use of Relative Importance Indices in Organizational Research. *Organ Res Methods*. 2004;7(3):238-257. doi:10.1177/1094428104266510
29. Azen R, Budescu D V. The Dominance Analysis Approach for Comparing Predictors in Multiple Regression. *Psychol Methods*. 2003;8(2):129-148. doi:10.1037/1082-989X.8.2.129

eTable 1. Sample characteristics of subset of mTBI comparison groups.

	Unit	Whole sample	Group differences		
			TBI-	mTBI+	P value
	N	554	394	160	
Sex					<0.001[a]
Female	N (%)	354 (63.9%)	273 (69.3%)	81 (50.6%)	
Male	N (%)	200 (36.1%)	121 (30.7%)	79 (49.4%)	
Age (in years)	mean ± SD	51.2 ± 5.5	51.4 ± 5.4	50.9 ± 5.8	0.33
Education (in years)	mean ± SD	16.8 ± 3.5	16.9 ± 3.6	16.7 ± 3.3	0.57
APOE4	N (%) positive	215 (38.8%)	153 (38.8%)	62 (38.8%)	1.00
Family history	N (%) positive	290 (52.3%)	213 (54.1%)	77 (48.1%)	0.24
Hypertension	N (%) positive	92 (16.6%)	59 (15.0%)	33 (20.6%)	0.14
Hyperlipidaemia	N (%) positive	68 (12.3%)	45 (11.4%)	23 (14.4%)	0.43
Diabetes Mellitus	N (%) positive	16 (2.9%)	11 (2.8%)	5 (3.1%)	1.00
BMI	mean ± SD	27.5 (5.14)	27.4 (5.1)	27.8 (5.3)	0.35
Current smoker	N (%) positive	29 (5.2%)	15 (3.8%)	14 (8.8%)	0.03*
High alcohol intake[c]	N (%) positive	78 (14.1%)	52 (13.2%)	26 (16.2%)	0.42
CVD risk[d]	mean ± SD	8.0 ± 3.9	7.8 ± 3.7	8.4 ± 4.2	0.17

Abbreviations: TBI, traumatic brain injury; BMI, body mass index; CVD, cardiovascular disease.
[a] p<0.001.
[b] p<0.05.
[c] High alcohol intake is defined as >21 units per week.
[d] Framingham Risk Score.

© 2024 Low A et al. *JAMA Network Open.*

eTable 2. Association between cardiovascular disease (CVD) risk and cerebral small vessel disease (SVD).

SVD markers [a]	95% CI	
	Whole sample (n=617)	TBI-/mTBI (n=554)
CMB count	-0.26, 0.36	-0.14, 0.56
Lacune count	0.09, 1.05	0.12, 1.14
WMH volume[b]	0.003, 0.02	0.001, 0.03
PVS-CSO	-0.04, 0.11	-0.04, 0.13
PVS-BG	0.002, 0.10	0.01, 0.11

Abbreviations: CI, confidence intervals; SVD, cerebral small vessel disease; mTBI, mild traumatic brain injury; CMB, cerebral microbleeds; WMH, white matter hyperintensities, PVS, perivascular spaces, CSO, centrum semiovale; BG, basal ganglia.
[a] Analyses were adjusted for sex, age, years of education, and study site.
[b] Normalised volume adjusted for total intracranial volume (TIV): (volume in mL/TIV) × 100%.

© 2024 Low A et al. *JAMA Network Open.*

eTable 3. Association between traumatic brain injury (TBI) and cerebral small vessel disease (SVD).

SVD markers	95% CI					
	All TBI (n=617)			Mild TBI (n=554)		
	Model 1 [a]	Model 2 [b]	Model 3 [c]	Model 1 [a]	Model 2 [b]	Model 3 [c]
CMB count	0.01, 0.18	0.02, 0.19	0.03, 0.20	0.02, 0.21	0.02, 0.21	0.04, 0.23
Lacune count	-0.07, 0.05	-0.07, 0.05	-0.07, 0.06	-0.09, 0.04	-0.09, 0.04	-0.09, 0.05
WMH volume[d]	-0.02, 0.02	-0.02, 0.02	-0.02, 0.02	-0.03, 0.02	-0.03, 0.02	-0.03, 0.02
PVS-CSO	-0.17, 0.09	-0.17, 0.08	-0.14, 0.12	-0.13, 0.15	-0.14, 0.14	-0.11, 0.19
PVS-BG	-0.05, 0.11	-0.05, 0.11	-0.05, 0.12	-0.03, 0.14	-0.03, 0.14	-0.02, 0.16

Abbreviations: CI, confidence intervals; SVD, cerebral small vessel disease; CMB, cerebral microbleeds; WMH, white matter hyperintensities, PVS, perivascular spaces, CSO, centrum semiovale; BG, basal ganglia.

[a] Model 1: Analyses were adjusted for sex, age, years of education, and study site.

[b] Model 2: Analyses were adjusted for sex, age, years of education, study site, and smoking.

[c] Model 3: Analyses were adjusted for sex, age, years of education, study site, smoking, APOE4 status, hypertension, hyperlipidaemia, diabetes mellitus, alcohol intake, overall Framingham CVD risk score.

[d] Normalised volume adjusted for total intracranial volume (TIV): (volume in mL/TIV) × 100%.

© 2024 Low A et al. *JAMA Network Open.*

eTable 4. Association between traumatic brain injury (TBI) and clinical features.

Clinical features	95% CI					
	All TBI (n=617)			Mild TBI (n=554)		
	Model 1 [a]	Model 2 [b]	Model 3 [c]	Model 1 [a]	Model 2 [b]	Model 3 [c]
Memory	-0.09, -0.004	-0.09, 0.0004	-0.1, -0.003	-0.07, 0.03	-0.07, 0.03	-0.08, 0.03
Language	-0.08, 0.03	-0.07, 0.03	-0.07, 0.04	-0.07, 0.04	-0.07, 0.04	-0.06, 0.05
Attention	-0.06, 0.02	-0.06, 0.02	-0.05, 0.04	-0.04, 0.04	-0.04, 0.05	-0.03, 0.06
Visuospatial	-0.09, 0.01	-0.08, 0.01	-0.08, 0.01	-0.09, 0.01	-0.09, 0.02	-0.09, 0.02
Depression[d]	0.33, 1.67	0.26, 1.61	0.31, 1.70	0.10, 1.52	0.10, 1.53	0.10, 1.57
Sleep[d]	0.18, 0.61	0.16, 0.60	0.17, 0.62	0.13, 0.61	0.14, 0.62	0.14, 0.64
Gait[d]	0.002, 0.03	0.001, 0.03	0.002, 0.03	0.003, 0.03	0.002, 0.03	0.002, 0.03

Abbreviations: CI, confidence intervals; TBI, traumatic brain injury.
[a] Model 1: Analyses were adjusted for sex, age, years of education, and study site.
[b] Model 2: Analyses were adjusted for sex, age, years of education, study site, and smoking.
[c] Model 3: Analyses were adjusted for sex, age, years of education, study site, smoking, APOE4 status, hypertension, hyperlipidaemia, diabetes mellitus, alcohol intake, overall Framingham CVD risk score.
[d] Statistically significant; 95% CI does not include zero.

© 2024 Low A et al. *JAMA Network Open.*
