# Supplementary material for: Neuroimaging and Clinical Findings in Healthy Middle-Aged Adults With Mild Traumatic Brain Injury in the PREVENT Dementia Study
Source: JAMA Netw Open. 2024 Aug 15;7(8):e2426774. doi: 10.1001/jamanetworkopen.2024.26774 (PMC11327885; doi:10.1001/jamanetworkopen.2024.26774)
Supplement: Supplement 2. — Nonauthor Collaborators [file jamanetwopen-e2426774-s002.pdf]

Supplemental Online Content: Nonauthor Collaborators
*First name, last name, and suffix (if applicable) are required and will appear in PubMed.

*Group Name(s): the PREVENT Dementia Investigators							
***First Name and Middle Initial(s)**	***Last Name**	***Suffix (eg, Jr, III)**	Academic Degrees	Institution	Location (city, state/province, country)	Role or Contribution, eg, chair, principal investigator	Group (if more than 1 Group listed in the byline) and/or Subgroup (eg, Steering Committee)
Katie	Bridgeman		BSc	Edinburgh Dementia Prevention, University of Edinburgh	Edinburgh, Scotland, UK	PREVENT National Coordinator	Steering Group
Leo	Chouliaras		MBBS PhD	Department of Psychiatry, University of Cambridge School of Clinical Medicine	Cambridge, Cambridgeshire, UK	Steering Group	
Siobhan	Coleman		BA	Windsor Research Unit, Cambridgeshire and Peterborough NHS Foundation Trust	Cambridge, Cambridgeshire, UK	Cambridge site Study Coordinator	
Hannah	Darwin		MSc	Edinburgh Dementia Prevention, University of Edinburgh	Edinburgh, Scotland, UK	Edinburgh site Research Coordinator	
Maria-Eleni	Dounavi		PhD	Department of Psychiatry, University of Cambridge School of Clinical Medicine	Cambridge, Cambridgeshire, UK	PREVENT researcher	
Feng	Deng		PhD	Global Brain Health Institute, Trinity College Dublin	Dublin, Ireland, UK	PREVENT researcher	
Robert	Dudas		MD, PhD, FRCPsych, PGCert Med Ed	Cambridgeshire and Peterborough NHS Foundation Trust	Cambridge, Cambridgeshire, UK	PREVENT researcher	
Sarah	Gregory		PhD	Scottish Brain Sciences; Edinburgh Dementia Prevention, University of Edinburgh	Edinburgh, Scotland, UK	PREVENT researcher	

Supplemental Online Content: Nonauthor Collaborators
*First name, last name, and suffix (if applicable) are required and will appear in PubMed.

***First Name and Middle Initial(s)**	***Last Name**	***Suffix (eg, Jr, III)**	Academic Degrees	Institution	Location (city, state/province, country)	Role or Contribution, eg, chair, principal investigator	Group (if more than 1 Group listed in the byline) and/or Subgroup (eg, Steering Committee)
Ivan	Koychev		PhD	Department of Psychiatry, University of Oxford	Oxford, Oxfordshire, UK	Principal Investigator	
Brian	Lawlor		MD, FRCPI, FRCPsych	Global Brain Health Institute, Trinity College Dublin	Dublin, Ireland, UK	Principal Investigator	Steering Group
Audrey	Low		PhD	Department of Psychiatry, University of Cambridge School of Clinical Medicine	Cambridge, Cambridgeshire, UK	PREVENT researcher	
Clare	Mackay		PhD	Department of Psychiatry, University of Oxford	Oxford, Oxfordshire, UK	Oxford site Imaging lead	
Elijah	Mak		PhD	Department of Psychiatry, University of Cambridge School of Clinical Medicine	Cambridge, Cambridgeshire, UK	PREVENT researcher	
Paresh	Malhotra		PhD	Department of Brain Sciences, Imperial College London	London, UK	Principal Investigator	Steering Group
Graciela	Muniz-Terrera		PhD	Edinburgh Dementia Prevention, University of Edinburgh; Ohio University Heritage College of Osteopathic Medicine	Edinburgh, Scotland, UK; Athens, Ohio, USA	Principal Investigator and Biostatistics lead	Steering Group
Lorina	Naci		PhD	School of Psychology and Global Brain Health Institute, Trinity College Dublin	Dublin, Ireland, UK	Principal Investigator	

Supplemental Online Content: Nonauthor Collaborators

*First name, last name, and suffix (if applicable) are required and will appear in PubMed.

***First Name and Middle Initial(s)**	***Last Name**	***Suffix (eg, Jr, III)**	Academic Degrees	Institution	Location (city, state/province, country)	Role or Contribution, eg, chair, principal investigator	Group (if more than 1 Group listed in the byline) and/or Subgroup (eg, Steering Committee)
John	O'Brien		DM	Department of Psychiatry, University of Cambridge School of Clinical Medicine; Cambridgeshire and Peterborough NHS Foundation Trust		Principal Investigator and Imaging lead	Steering Group
Vanessa	Raymont		MRCPsyc h	Department of Psychiatry, University of Oxford	Oxford, Oxfordshire, UK	Steering Group	
Craig	Ritchie		MD	Scottish Brain Sciences; Edinburgh Dementia Prevention, University of Edinburgh; Mackenzie Institute, St Andrews University	Edinburgh, Scotland, UK	Chief Investigator	Steering Group
Li	Su		PhD	Department of Psychiatry, University of Cambridge School of Clinical Medicine	Cambridge, Cambridgeshire, UK	PREVENT researcher	
Peter	Swann		MRCPsyc h	Department of Psychiatry, University of Cambridge School of Clinical Medicine; Cambridgeshire and Peterborough NHS Foundation Trust	Cambridge, Cambridgeshire, UK	PREVENT researcher	
Tony	Thayanandan		MSc	Department of Psychiatry, University of Oxford	Oxford, Oxfordshire, UK	PREVENT researcher	
Guy	Williams		PhD	Wolfson Brain Imaging Centre and Department of Clinical Neurosciences, University of Cambridge School of Clinical Medicine	Cambridge, Cambridgeshire, UK	Imaging protocol design	
