# Supplementary material for: Neuroimaging and Clinical Findings in Healthy Middle-Aged Adults With Mild Traumatic Brain Injury in the PREVENT Dementia Study
Source: JAMA Netw Open. 2024 Aug 15;7(8):e2426774. doi: 10.1001/jamanetworkopen.2024.26774 (PMC11327885; doi:10.1001/jamanetworkopen.2024.26774)
Supplement: Supplement 3. — Data Sharing Statement [file jamanetwopen-e2426774-s003.pdf]

Data Sharing Statement

Low. Neuroimaging and Clinical Findings in Healthy Middle-Aged Adults With Mild Traumatic Brain Injury. *JAMA Netw Open*. Published August 15, 2024.
doi:10.1001/jamanetworkopen.2024.26774

Data
Data available: Yes
Data types: Deidentified participant data
How to access data: The PREVENT dataset is available to access through a data request on the study website (www.preventdementia.co.uk); on the Alzheimer's Disease Data Initiative (ADDI) platform baseline dataset DOI: https://doi.org/10.34688/PREVENTMAIN_BASELINE_700V1; Dementia Platforms UK (DPUK); and the Global Alzheimer's Association Network (GAAIN).
When available: With publication

Supporting Documents
Document types: None

Additional Information
Who can access the data: Data will be made available to researchers whose proposed use of the data has been approved by the PREVENT Dementia programme steering committee.
Types of analyses: Data will be made available for the purpose of the proposed analysis.
Mechanisms of data availability: After approval of a proposal and with a data access agreement.
